# Autism Spectrum Disorder in Children Is Not Associated With Abnormal Autonomic Nervous System Function: Hypothesis and Theory

**DOI:** 10.3389/fpsyt.2022.830234

**Published:** 2022-03-15

**Authors:** Ashley Barbier, Ji-Hong Chen, Jan D. Huizinga

**Affiliations:** Department of Medicine, McMaster University, Hamilton, ON, Canada

**Keywords:** parasympathetic and sympathetic reactivity, autism (ASD), gastrointestinal disorders, respiratory sinus arrhythmia (RSA), autonomic nervous system, heart rate variability (HRV), electrodermal activity

## Abstract

The quest to understand the pathophysiology of autism spectrum disorder (ASD) has led to extensive literature that purports to provide evidence for autonomic dysfunction based on heart rate and heart rate variability (HRV), in particular respiratory sinus arrhythmia (RSA), a measure of parasympathetic functioning. Many studies conclude that autism is associated with vagal withdrawal and sympathetic hyperactivation based on HRV and electrodermal analyses. We will argue that a critical analysis of the data leads to the hypothesis that autonomic nervous system dysfunction is not a dominant feature of autism. Most children with ASD have normal parasympathetic baseline values and normal autonomic responses to social stimuli. The existing HRV and electrodermal data cannot lead to the conclusion of an over-excitation of the sympathetic nervous system. A small subgroup of ASD children in experimental settings has relatively low RSA values and relatively high heart rates. The data suggest that this is likely associated with a relatively high level of anxiety during study conditions, associated with co-morbidities such as constipation, or due to the use of psychoactive medication. Many studies interpret their data to conform with a preferred hypothesis of autonomic dysfunction as a trait of autism, related to the polyvagal theory, but the HRV evidence is to the contrary. HRV analysis may identify children with ASD having autonomic dysfunction due to co-morbidities.

## Introduction

Autism spectrum disorder (ASD) is a neurodevelopmental condition that affects social communication and social interaction. Heterogeneity of the condition results in a broad spectrum of presentations through symptoms and levels of functioning. ASD is often characterized by restrictive and repetitive patterns of behavior (1–3) and atypical social interactions (e.g., non-verbal behaviors, eye-gaze, and facial expressions) (4). Porges (5) proposed the idea that children with ASD are unable to display appropriate psychophysiological flexibility in response to stimuli due to autonomic inflexibility and chronic sympathetic activation. Individuals with ASD are described as in a chronic state of hyperarousal (6). The polyvagal theory proposes that a functional “vagal brake”, or spontaneous engagement and disengagement of the myelinated vagus based on environmental risk, is associated with behavioral flexibility and lowered vulnerability to stress (5, 7). It is suggested that children with ASD do not execute this vagal brake, therefore, they do not show autonomic flexibility to stimuli. The polyvagal theory indicates that this is due to dysfunction of the neuroception of a threat, leading to chronic vagal withdrawal and decreases in parasympathetic activity, specifically to unfamiliar social stimuli (8).

The measurement of respiratory sinus arrhythmia (RSA), a parasympathetic parameter of heart rate variability, is integral to the polyvagal theory, no doubt the reason why many studies on autonomic functioning in ASD often exclusively measure RSA. RSA is proposed as a portal, allowing accurate measurement of the dynamic influence of myelinated vagal efferent pathways onto the sino-atrial node; specifically, the communication between the nucleus ambiguous and the heart. The nucleus ambiguous is also critical for esophageal function and facial expressions associated with emotion (9). Although there are several mechanisms to modulate heart rate, only the myelinated vagal efferent pathways from the nucleus ambiguous *via* nicotinic preganglionic receptors on the sino-atrial node are proposed to be capable of the rapid, instantaneous changes that characterize RSA (10). Efferent projections from the nucleus ambiguous are involved with processes associated with feeding and breathing, facial movements to express emotion, and to communicate internal states in a social context; RSA is proposed to measure this neuronal traffic (9).

Our objective was to evaluate the literature that *measured* features of the autonomic nervous system such as heart rate variability and electrodermal activity to evaluate the evidence of autonomic dysfunction in ASD.

## Assessing Autonomic Dysfunction in ASD *via* Heart Rate and Heart Rate Variability

Heart rate can react momentarily to changes in nervous input from the autonomic nervous system, and this property establishes heart rate variability (HRV) as a mirror of autonomic activity (11–13). HRV has been widely used to evaluate autonomic functioning, not only pertaining to cardiac function but to many other physiological and psychological aspects of body functioning (11, 14, 15). HRV does not reflect exclusively cardiac control systems. For example, the parasympathetic regulation of breathing influences blood pressure and the subsequent activation of baroreceptors influences heart rate variability (16, 17). Hence, the autonomic regulation of breathing is seen in HRV. It is important to realize that the different HRV parameters for sympathetic or parasympathetic activity will not reflect all autonomic activity occurring throughout the body. Organs have distinct sympathetic and parasympathetic neuronal circuitries and activities that may or may not directly or indirectly influence HRV. Although there are many potential HRV parameters that can be evaluated, most autism studies employ heart rate and RSA (18). Although the sympathetic nervous system is considered relevant to ASD and the polyvagal theory, it is usually not assessed, and if assessed, electrodermal activity is the dominant technique used.

### General Conclusions Found in the Literature Based on Heart Rate and HRV Analysis

All studies that use HRV as a measure of autonomic functioning in ASD link their findings to the polyvagal theory, but few provide actual raw data on HRV parameters which was also noted in a review by Benevides and Lane (19). High baseline RSA is thought to be associated with adaptive social functioning (20), while low baseline RSA is believed to be associated with stress (21) and emotional dysregulation (22). In socially safe contexts, heart rate is thought to decrease due to vagal activity from the nucleus ambiguous acting on the heart and promoting appropriate social behavior (7). Studies evaluating ANS functioning in ASD suggest chronic sympathetic activation and vagal withdrawal in autism due to findings of lower RSA and higher heart rate at baseline and in response to stimuli, compared to controls (22–24). When studies report lower RSA and higher heart rate averages in both adults and children with ASD compared to a control group, the conclusion is that individuals with ASD exhibit chronic mobilization and impairment of the soothed autonomic state (8, 22). It is suggested that children with ASD have inaccurate nervous system perception when assessing risk, preventing the inhibition of limbic structures for immobilization and resulting in chronic vagal withdrawal (8, 24). We will argue that the perceived attractiveness of the polyvagal theory leads many authors to conclude that their HRV results are consistent with the theory, despite their data indicating otherwise.

## Critical Analysis of HRV Parameter Assessments

### Do Children With ASD Have an Abnormal Baseline RSA?

**Figure 1:**
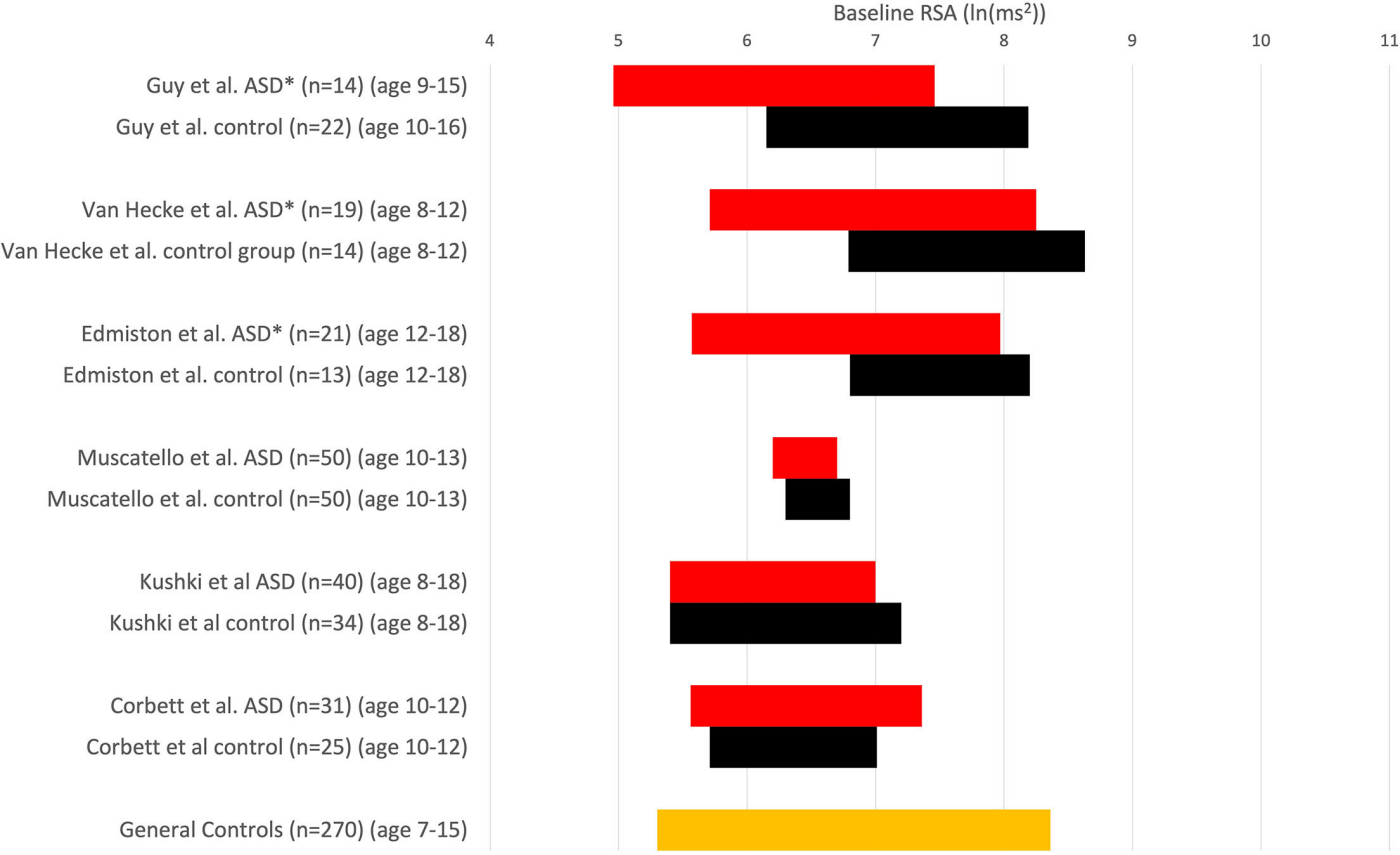
RSA is expressed in comparative studies with an ASD group (red) and typically developing children (control, black). RSA ± 1SD; *P < 0.05 compared to in-study controls. Others: no significant difference. Note that comparisons cannot be absolute because of differences in measuring RSA. Muscatello et al. and Corbett et al. used ln(HF power) with the HF range of 0.12–0.40 Hz, Kuski et al. (27) used 0.24–1.04 Hz. Edmiston et al. (23) used 0.15–0.40 Hz. Guy et al. (22) report that the amplitude of RSA was calculated as the natural logarithm of the extracted variance for each successive 30-s epoch within 12–1 Hz (probably 0.12–1 Hz). Vaughan van Hecke et al. (24) chose the natural logarithm of the variance of the band-pass series from HF, 0.12–1 Hz. At the bottom, the orange control values are derived from a study that examined RSA over time in 270 children (28); we used a range based on their average values ± 1 SD from ages 7–15, obtaining a normal range of 5.3–8.4 ln(ms^2^).

Despite numerous assertions in the literature to the contrary, the absolute values of baseline RSA of children with ASD are almost all within the normal range. The wide range of normal RSA values in children was documented by Harteveld et al., who reported on 4,822 children aged 0.5–20 years (25). Harteveld et al. (25) used the peak-valley method, measuring RSA by subtracting the shortest inter-beat interval during inhalation from the longest inter-beat interval during exhalation, and found that for 328 children aged 13–15, the RSA ranged from 18.7 to 186.7 ms (2.5–97.5 percentile), hence a very wide normal range. In a personal communication, Harteveld calculated the range of RSA values (2.5–97.5 percentile) in 99 typically developing children, aged 4–18, to be 4.5–8.8 ln(ms^2^) (Table 1). Dollar et al. studied 270 children and followed them from 2 to 15 years. For ~200 children, the RSA at 10 and 15 years ranged from 5.5 to 7.8 ln(ms^2^) (Figure 1) (28).

**Table 1:** Control values for RSA in a cohort of healthy children from a study by Nederend et al. (26) as shown in Harteveld et al. (25).

**Age**	**RSA, ln(ms** ^**2**^ **)**											
	**Boys**						**Girls**					
	***N***	**Median**	**Percentile**		**Mean**	**SD**	***N***	**Median**	**Percentile**		**Mean**	**SD**
			**2.5th**	**97.5th**					**2.5th**	**97.5th**		
1–3	8	5.01	4.68	6.42	5.42	0.74	10	5.34	3.33	7.28	5.26	1.27
4–7	17	7.62	5.06	8.63	7.13	1.12	6	7.09	6.43	8.74	7.39	0.94
8–10	8	7.70	5.53	8.63	7.54	1.06	12	7.07	4.45	8.84	6.73	1.44
11–12	9	7.16	5.94	9.01	7.35	1.05	8	7.50	5.72	8.01	7.38	0.79
13–15	11	7.03	5.02	7.69	6.90	0.78	13	7.22	5.82	9.31	7.32	1.06
16–18	8	6.54	5.45	7.19	6.61	0.58	7	6.02	4.74	8.33	6.37	1.23

*Data was obtained through personal communication with Dr. L. M. Harteveld with permission*.

When the values of heart rate or RSA of a cohort of children with ASD are compared to neurotypical children, the average values can show statistically significant differences. However, almost all children with ASD in those studies have values within the normal range, *even when compared to the control group of that study* (Figure 1). If most children with ASD have values within the range of control values, one cannot make the general statement that children with ASD have abnormal RSA values, implying autonomic dysfunction (29). Suppose some children with ASD fall outside of chosen confidence levels, say outside the 95% confidence interval. In that case, a subgroup may be the reason for the significant difference from the control group, and the existence of a subgroup may be highly clinically significant. It is well known that ASD has heterogeneous pathophysiology. Once a statistically significant difference is found, the reason for it should be established. Then the question ought to be whether the difference is physiologically and clinically significant or relevant. Hence, the statement that “a group of children with ASD have a statistically significant lower RSA baseline compared to a group of normally developing children” is not equivalent to the statement that “children with ASD have a low RSA baseline” and most certainly not that “children with ASD have autonomic dysfunction.”

Kushki et al. (27) and Muscatello et al. (30) did not observe baseline RSA differences. Corbett et al. reported that children with ASD did not show poor autonomic regulation during social interaction with novel peers, based on similar RSA values (31). Vaughan van Hecke et al. (24) reported baseline RSA to be significantly lower in an ASD group compared to typically developing controls. While statistically significant, the RSA values in the group of children with ASD fall within the normal range of control values (25), and most values also fall within the experimental control group values (Figure 1); hence their conclusion that “ASD patients have a lower baseline RSA” is not correct. The only valid conclusion is that a small subgroup of patients with RSA values falls outside the control group's confidence levels (or average ± one standard deviation range).

Edmiston et al. (23) recorded baseline RSA values in ASD children, finding that the average value was different from that in controls (Figure 1) and concluded that children with ASD have “reduced physiological self-regulation.” This conclusion must be rejected since the average RSA value of children with ASD, between 12 and 18 years, was ~6.9 ln(ms^2^) which is entirely normal. There are no data that show that an RSA of 6.9 ln(ms^2^) constitutes autonomic dysfunction. Bal et al. (32), based on RSA baseline values, stated that children with autism have “lower overall vagal regulation of the heart”; this is not only incorrect, since the absolute baseline values fall within the overall normal range, it is also misleading to suggest that something is wrong with regulation of cardiac function. Neuhaus correlated baseline RSA values with autism features, but the average baseline RSA of 6.9 ln(ms^2^) in the children with autism cannot be interpreted as indicating autonomic dysfunction (33). Miller tried to relate baseline RSA values, which are mainly normal, with features of autism, but no linear relationships were found (34, 35).

It is illustrative to point out that when normal findings are found, they are often judged to be unreliable, indicating the desire to find dysfunction. For example: “Adults with autism demonstrated significantly higher baseline HRV [using the root mean square of successive differences (RMSSD)] compared to control groups (36) possibly suggesting effective interventions/supports to develop control over their physiological state or, alternatively, that higher RMSSD values may have been inflated due to movement, or heart rate and respiratory influences” (8). Zahn et al. concluded that none of the variables to index the construct of autonomic nervous system arousal was significantly different from controls (36). When Smeekens et al. did not find any differences in autonomic or endocrine activity with social functioning in adults, it was thought to be due to lack of power (37), and a non-significant effect was worded as a “blunted increase.”

Bricout et al. (38) used a clinically prominent test for autonomic dysfunction and found that children with ASD did not have clinical signs of dysautonomia in response to the head-up tilt test. As reflected by RMSSD, their baseline parasympathetic tone was also not different from controls. In a case series of 6 patients with ASD who had symptoms of autonomic dysfunction: postural lightheadedness, near syncope, constipation, diarrhea and early satiety, all had postural tachycardia, but no orthostatic hypotension (39). The absence of orthostatic hypotension is interesting since it is a common feature of neurodegenerative disorders (40).

### Do Children With ASD Have an Abnormal RSA in Response to Stimuli?

Assessment of RSA reactivity to stimuli is probably the most relevant experimental condition to be studied concerning potential autonomic dysfunction related to ASD in the context of HRV. This has been assessed in two ways: analysis of differences in absolute values of RSA during the experimental conditions (Figure 3) and reactivity, the difference between baseline and experimental condition. In response to a stressful mental load, HRV shows a consistent decrease, including a decrease in RSA (35). Hence a decrease would show autonomic flexibility.

**Figure 2:**
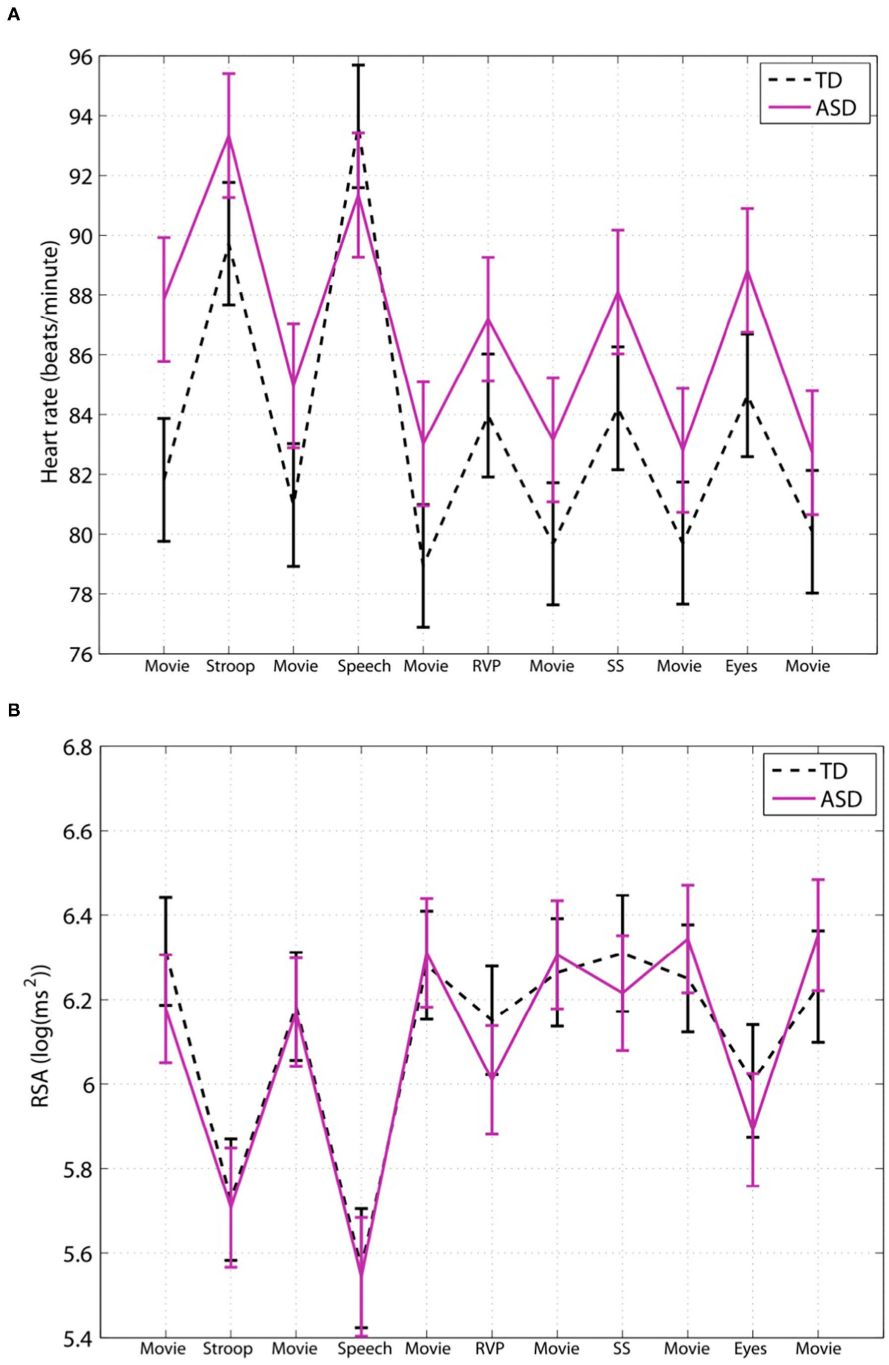
Children with ASD have a normal baseline HRV parameter and a normal autonomic response to social stimuli. This figure is taken from a study by Kushki et al. (27); they studied autonomic regulation in children with autism while performing tasks that elicit anxiety, attention, response inhibition and social cognition. Expressed are heart rate **(A)** and RSA **(B)**. The authors conclude that children with ASD show overall autonomic hyperarousal and selective atypical reactivity to social tasks. **(A)** The average baseline heart rate of the children with ASD was 88 bpm, which is not indicative of cardiac dysfunction nor an overactive sympathetic nervous system; it cannot be interpreted to show that children with ASD show hyperarousal. **(B)** There were no statistical differences in baseline RSA nor general group differences. Evaluating each task, there were no differences in RSA reactivity in the Stroop, public speaking or rapid visual information processing tasks. The reading the mind in the eyes task also did not show a significant difference except when the medication group was excluded. However, both the control group and the ASD children showed a normal strong decrease in RSA, and there is no evidence that this difference is clinically significant. (TD), *n* = 34, and ASD children, *n* = 40. “Movie”: considered resting baseline; “Stroop”: eliciting a stress reaction; “Speech”: public speaking considered anxiety eliciting; “RVP”: rapid visual information processing, eliciting sustained attention; “SS”: stop-signal task, testing response inhibition; “Eyes”: reading the mind in the eyes, testing social cognition. This figure labels RSA to be log(ms^2^) however the values indicate that RSA is likely ln(ms^2^).

Figure 2 shows data from an important study by Kushki et al. (27). The data pertaining to baseline RSA and RSA reactivity and baseline heart rate and heart rate reactivity to various stimuli leave us with only one logical conclusion, that ASD children have normal autonomic functioning. Tasks are accompanied by an increase in heart rate and a decrease in RSA, similar to controls, consistent with the author's results statement: “After controlling for age, sex and full-scale IQ, the performance of children in the ASD group was not significantly different from that of the typically developing control group on any of the five tasks in this study.” Yet, the authors state: “In the absence of such differences, ANS atypicalities may suggest compensatory mechanisms applied by the ASD group” (27). A simpler and likely better explanation is that the ASD children do not show any clinically significant autonomic dysfunction. The desire to find differences can be deduced from this statement: “Our results suggest atypical cardiac findings… in particular, while not statistically significant, we found that the ASD group had an elevated heart rate during the experimental session” (27). Hence, even though there was no statistical difference between children with autism and the control group, this non-difference is suggested to be an “atypical cardiac finding.” The conclusion of the authors that “ASD children show selective atypical reactivity” does not appear to have clinical relevance.

**Figure 3:**
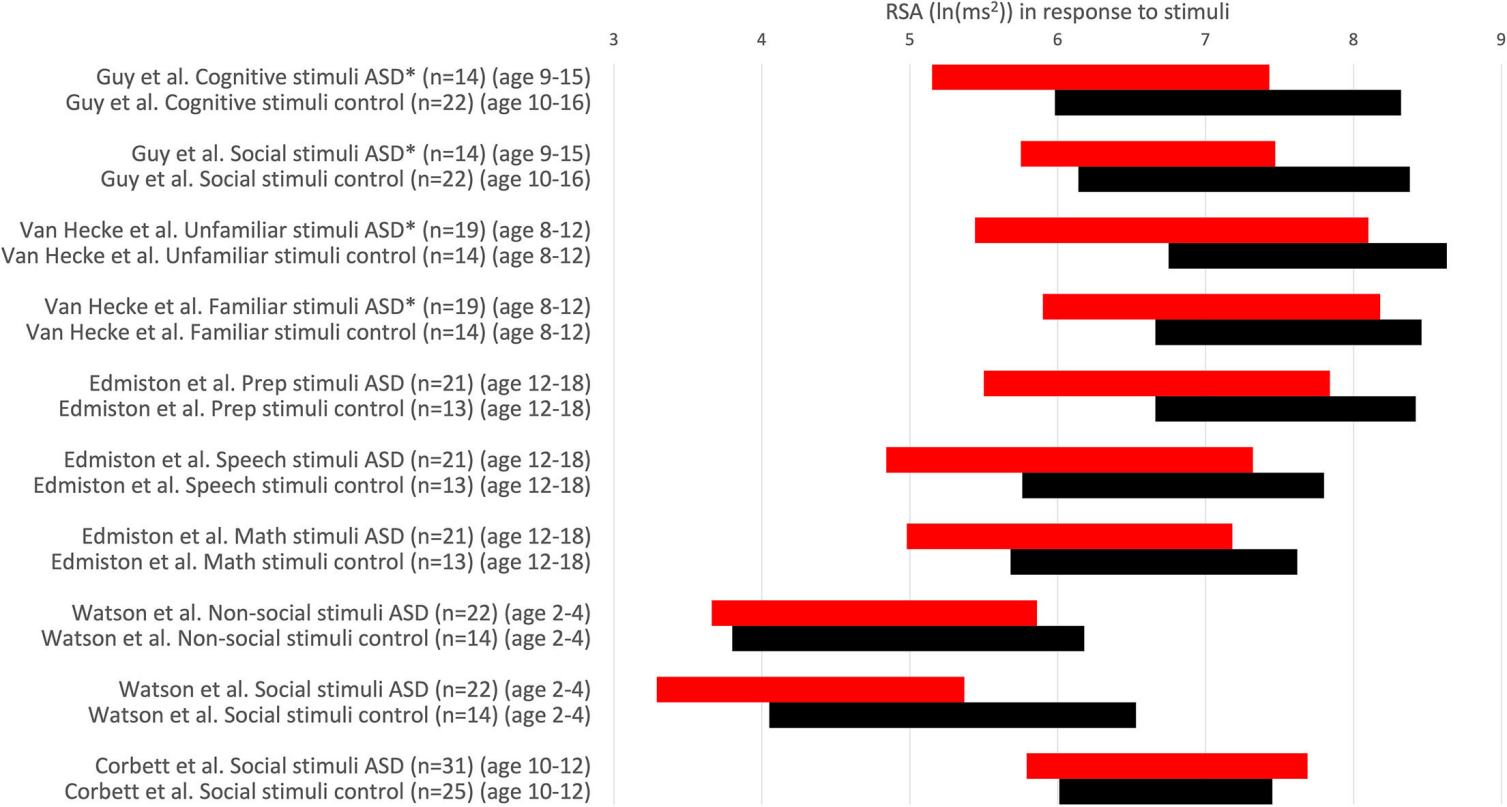
RSA values in response to stimuli. RSA values are expressed comparing the response to stimuli in children with ASD (red) and a control group (black). RSA ± 1SD. *P < 0.05. Others: no significant difference. RSA is expressed in ln(ms^2^).

Guy et al. (22) conducted a study investigating the RSA response to cognitive and social stimuli in children with ASD compared to age- and IQ-matched typically developing controls. The study reports that ASD is associated with abnormal HRV (lower RSA) across all tasks, corroborating the polyvagal theory and concluding that low RSA in autism is due to less parasympathetic activity and vagal withdrawal. While differences in reported RSA values are statistically significant, the absolute values of RSA in ASD groups (Figure 3) [6.29 ln(ms^2^) during a cognitive task; 6.61 ln(ms^2^) during a social task] fall categorically within the normal range of typically developing controls. Hence, the conclusion of abnormal RSA in children with ASD is not supported. The discussion states that all findings could be related to anxiety, and yet it is stated that the data support the use of HRV as a biomarker for ASD (22).

Muscatello et al. (30) exposed participants to a social interaction protocol, the Trier Social Stress Test-Friendly (TSST-F). The data show that “ASD and typically developing youth did not differ in mean RSA or RSA responsivity during the TSST-F paradigm when controlling for age.” Instead of giving the study the title that no evidence of autonomic dysfunction was found, the title became “Evidence for decreased parasympathetic response to a novel peer interaction in older children with autism spectrum disorder.” The term “decreased parasympathetic” does not appear in the study itself; instead, the term “blunted” is used; however, no statistical differences were found.

Toichi and Kamio (35) studied high functioning young adults with ASD to avoid confounding factors such as the inability to sit still. They did not find any differences with a control group related to heart rate, sympathetic activity or resting parasympathetic activity. The parasympathetic activity (measured based on the Lorenz plot) decreased in 18/20 controls with no change in 2/20. The ASD group showed a decrease in 10/20, 3/20 did not show a response and 7/10 showed an increase, although this increase was not repeated when other types of mental activity were examined. The authors suggest several explanations for an increase in parasympathetic activity. Some persons with ASD might find mental tasks relaxing, or it may be related to the functioning of the amygdala (35). Interestingly, Muscatello et al. (30) studied the effect of social interactions to promote a relaxing environment. Indeed, the RSA increased, but did so similarly in the control and the ASD group. The increase in RSA in both the control and the ASD group in the study by Guy et al. related to a social task that involved a positive social engagement with an adult clinician (22).

Edmiston et al. (23) concluded that social problems in ASD may be linked to the RSA response to social stress; however, the RSA response to the stress was the same as in controls, a reduction of ~1 unit ln(ms^2^). The absolute values of RSA during social judgment were statistically different from the control group. Still, the response was the same, and the absolute values were also normal and strongly overlapping.

Watson et al. (41) showed that RSA findings for children with ASD who had no or limited expressive language showed no significant difference from control groups in response to both non-social and social stimuli, concluding that their findings do not show that ASD participants have an underactive parasympathetic nervous system or disproportional arousal when attending to social vs. non-social stimuli.

Bazelmans et al. (42) showed that watching naturalistic videos did not show differences in heart rate or RSA between ASD and typically developing children and concluded that HRV did not produce biomarkers for ASD. In autistic adults, no differences in autonomic or endocrine parameters were found in response to a social interaction with an unfamiliar person, compared to a control group (37, 43).

A recent meta-analysis incorporating most studies reported here states that their analysis supports low HRV as a potential biomarker of ASD without critically evaluating the studies (44). Their bias was formulated in the introduction: “ASD feature stereotyped thought, behavior and problems of social interaction, therefore the connection between individuals with ASD and low HRV should be intuitive.” The study concludes that HRV does not differentiate ASD from other psychiatric disorders and suggests that results and conclusions should be viewed cautiously because co-morbidities might affect HRV, and this was not accounted for (44). Hence, the conclusion that HRV is a biomarker for ASD is not warranted.

### Do Children With ASD Have an Increased Abnormal Basal Heart Rate?

In many studies, the average baseline heart rate value in a cohort of children with ASD is higher compared to the control group. However, most ASD children have a heart rate that falls not only into the general normal heart rate range (45), it also falls within the range of normal values of the controls in the study, indicating that most ASD children have a normal heart rate from any perspective. Throughout the ASD literature, all heart rates are obtained in an experimental condition. Hence, if some children with ASD feel a higher level of anxiety in an experimental setting (46), the average heart rate of children with ASD in that group would likely be higher than the average heart rate of controls. The experimental conditions of studies are rarely accounted for. Yet, they are a valid factor that may account for higher averages of heart rate often seen in ASD groups, despite most individual ASD participants showing normal heart rate values. When a group of ASD children is compared to a group of typically developing controls, and the a*verage* heart rate value is higher in the ASD group, but most children in the ASD group have a normal heart rate, one can conclude that “the autism group has a significantly increased heart rate compared to the control group,” but one cannot conclude that “children with autism have an increased heart rate” and one can definitely not make the general statement that “children with ASD show sympathetic hyperarousal.”

**Figure 4:**
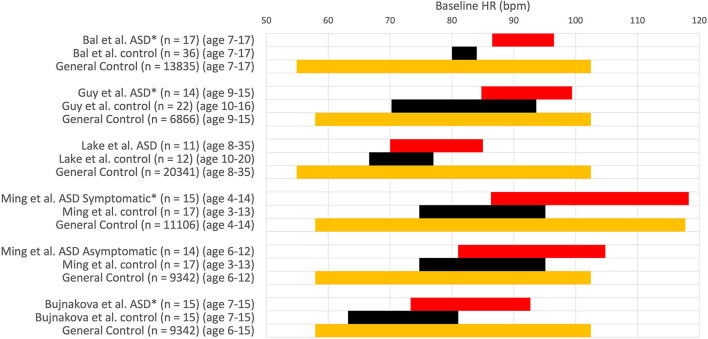
Heart rate values (HR ± 1SD) at baseline. HR expressed in comparative studies with an ASD group (red) and a group with typically developing children (control, black) and normal HR values in a large of children taken from Ostchega et al. (orange) (45). *P < 0.05. Others: no significant difference.

Bal et al. (32) conclude that children with ASD have significantly faster baseline heart rate than typically developing controls. While the ASD group's mean heart rate value was significantly higher than the typically developing control group, the heart rate of the ASD group was still within the normal range of the age demographics (45) as shown in Figure 4. The study concludes that ASD children had “lower overall vagal regulation of heart rate” and “hyperactive sympathetic activity” (32). These conclusions cannot be supported as the children with ASD have a clinically normal heart rate. A simple statement about sympathetic activity should also not be made based on heart rate alone, without measuring sympathetic activity directly. Kushki et al. (27) concluded that children with ASD have “overall autonomic hyperarousal” based on a “marginally elevated basal heart rate.” The average baseline heart rate of the children with ASD was 88 bpm, which is not indicative of cardiac dysfunction nor an overactive sympathetic nervous system (see Figure 4).

Bujnakova et al. (47) concluded that a higher heart rate at baseline in children with ASD compared to the age-matched control group indicated tachycardia; however, an average heart rate of 83 bpm in 7–15-year-old children does not indicate tachycardia. The heart rate values in this study are within the normal range of its age and gender demographics (45); hence it is unreasonable to conclude autonomic deficits based on these baseline heart rate data. The authors do mention the questionability of their findings related to comorbid psychiatric symptoms.

When children with ASD were grouped into those with known autonomic dysfunction (gastrointestinal motility problems or syncope, etc.) and those without, the heart rate and blood pressure were similar in controls and asymptomatics. They increased in symptomatics suggesting the higher heart rate to be due to comorbidity and not autism *per se* (48).

A group of 116 controls and 154 children with ASD showed an average heart rate of 90.1 and 95.2 bpm, respectively, which was significantly different; however, when only the 82 non-medicated ASD children were assessed, there was no difference with the control group (43).

### Do Children With ASD Have an Abnormal Increase in Heart Rate in Response to Stimuli?

Many studies in the autism literature find a higher heart rate in ASD groups at baseline, which remains consistent throughout exposure to stimuli, but this does not constitute a higher heart rate response. Kushki et al. (27) found no significant group differences for heart rate reactivity in the Stroop, Rapid Visual Information Processing, Stop Signal, or Reading the Mind in the Eyes tasks (Figure 2). The authors report blunted heart rate reactivity to social anxiety tasks due to reduced responsivity to the public speaking task; however data pertaining to heart rate responsivity in each task suggest excellent autonomic reactivity (Figure 2). They conclude atypical heart rate reactivity to social tasks, despite the increase in heart rate seen in both the ASD and control groups for all stimuli, which suggests that no autonomic dysfunction is present in the ASD group. Sheinkopf et al. (18) found that the ASD and control groups did not differ in mean heart rate during all stimulus conditions. With “distal stranger” stimuli, both groups had a heart rate that remained approximately the same, whereas with “proximal stranger” stimuli, both groups had decreased heart rate with no differences shown among groups.

**Figure 5:**
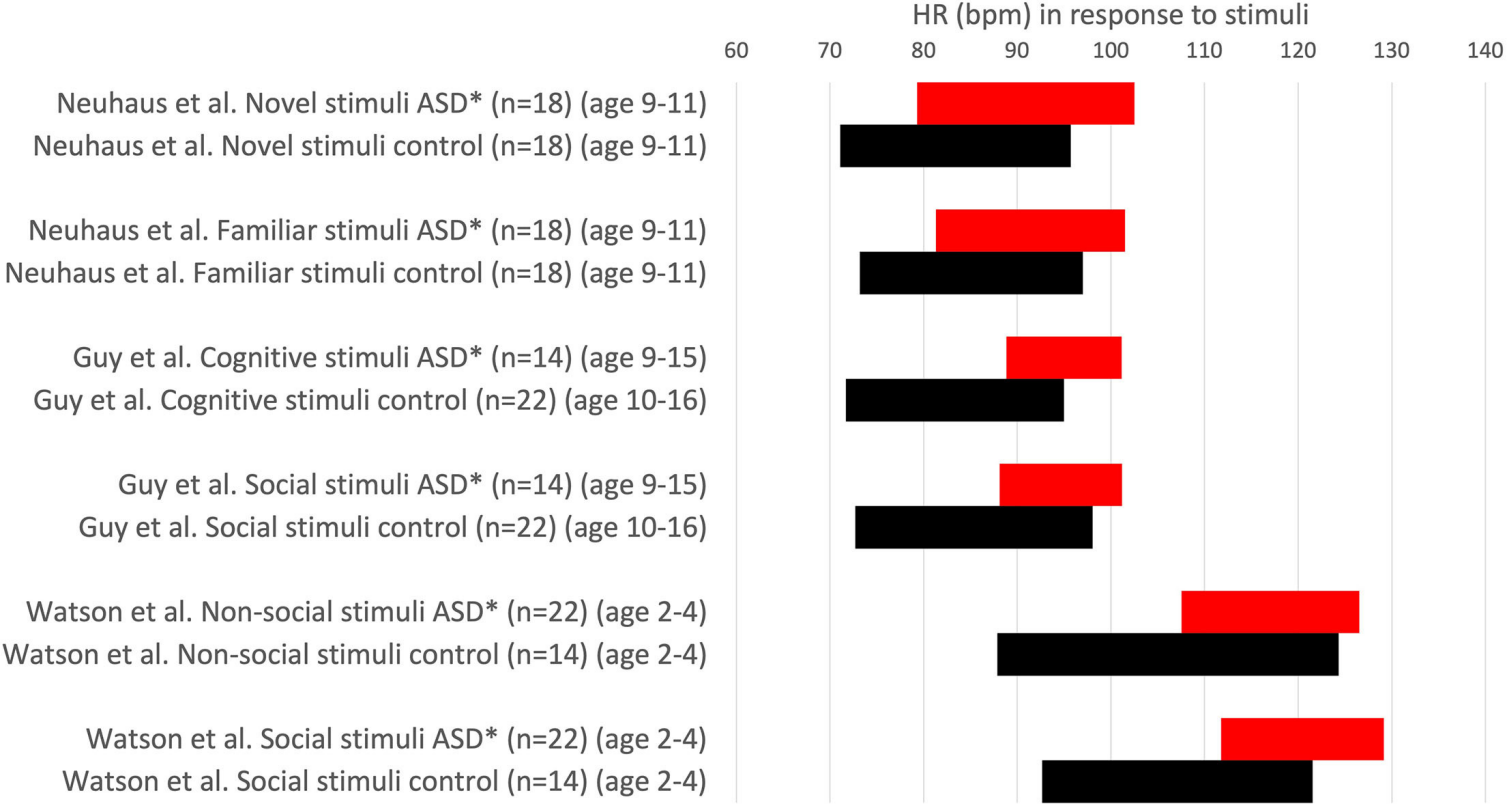
Heart rate values (HR ± 1SD) in response to stimuli. HR is expressed in comparative studies with an ASD group (red) and typically developing children (control, black). *P < 0.05. Others: no significant difference.

Neuhaus et al. (49) found that, in contrast to their expectations, the children with ASD had a normal heart rate and RSA response to interactions with a novel partner, indicating typical autonomic reactivity (Figure 5).

Watson et al. (41) found that an ASD group had a faster heart rate than age-matched controls, but heart rate was not specific to stimulus type for non-social and social stimuli. They conclude that their data do not support an underactive parasympathetic system nor disproportionate arousal when attending social stimuli. No differences in RSA in response to stimuli were observed between the ASD group and controls.

### Can Potential Parasympathetic Autonomic Dysfunction in ASD Be Assessed Using the Pupillary Light Reflex?

The pupillary light reflex expresses the constriction and subsequent dilation of the pupil in response to light as a result of the antagonistic actions of the iris sphincter and the dilator muscles (50). Latency and constriction are under parasympathetic control. Subsequent relaxation is due to sympathetic inhibition of parasympathetic neurons at the Edinger-Westphal nucleus as well as sympathetic contraction of the iris dilator muscle (50). Nyström et al. concluded that “infants at risk for autism have a hypersensitive pupillary light reflex,” suggested to be due to cholinergic autonomic “disruptions”; however, most infants had latency and constriction amplitudes that fell within the range of the control values (51). Daluwatte et al. showed that children with ASD, on average, have significantly longer latency and reduced pupil constriction amplitude in response to light compared to typically developing children; the difference in reflex parameters was suggested to be due to parasympathetic dysfunction, but it was not accompanied by a significant difference in RMSSD values (43). Fan et al. studied a group of children and young adults with ASD (52). They showed that the pupillary light reflex features, in particular the reflex latency, could discriminate between the ASD group and a control group, particularly the reflex latency. Lower constriction velocities were found in children with ASD compared with the typically developing control group, but this was statistically significant only at a light-adapted reflex stimulus intensity of 872 cd/m^2^ and not at other intensities; the ASD group exhibited significantly smaller relative constriction at a dark-adapted reflex stimulus intensity of 794 cd/m^2^ but not at other intensities (52). There was no statistical difference in the reflex recovery velocity between the two groups (52). Hence, children with autism have a robust pupillary light reflex. They may have a response that is not different from typically developing children, or they may have a response that is different in some features but not in other characteristics. The question is whether such a difference indicates pathophysiology, and if so, how this may correlate with autonomic functioning related to ASD traits or comorbidities.

## Assessment of Sympathetic Activity

The polyvagal theory predicts that children with autism would have a “hyper-responsive sympathetic system” (32, 53). To support this theory, autistic children should have a high sympathetic tone and exaggerated sympathetic responses to stress-provoking stimuli. An often-cited study by Hirstein et al. (54) makes strong statements about sympathetic autonomic dysfunction in children with autism, but it is dominated by discussion, with very few study data presented, and hence should be interpreted with caution. Hirstein et al. hypothesize that amygdala damage in children with autism causes disastrous brain malfunctions. They hypothesize that autistic children have chronic high sympathetic activity that they try to reduce by calming activities such as repetitive behaviors and that “autistic children use overt behavior in order to control a malfunctioning autonomic nervous system,” but no data to support this are provided.

Most studies on sympathetic activity in children with ASD use electrodermal activity. Electrodermal activity is defined as the electrical conductivity between two electrodes on the skin over time; it provides an index of sympathetic nervous system activity since eccrine sweat glands are innervated by the sympathetic but not parasympathetic branch of the autonomic nervous system (55). Although the study of electrodermal activity appears to be a logical non-invasive choice, there is no direct evidence that whatever brain sympathetic activity we are interested in is faithfully captured by electrodermal activity, but it might. The expectation is that during mental exercises, the sympathetic nervous system will be activated to respond to energy demand to increase blood glucose. If an exercise were accompanied by marked anxiety, this would further increase sympathetic activity.

**Figure 6:**
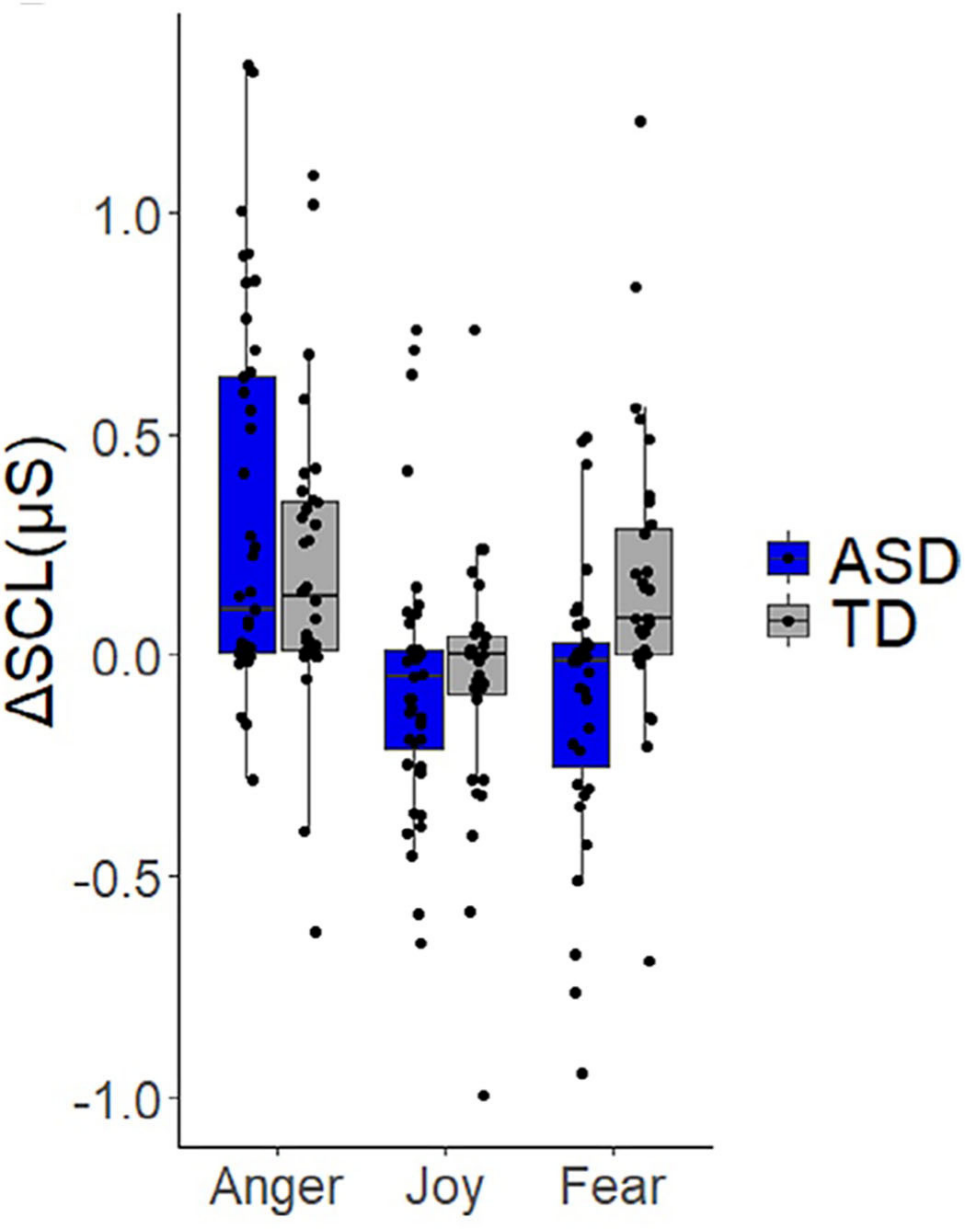
Children do not show a significant difference in sympathetic response to stimuli. Expression of skin conductance as a measure of activity in the sympathetic nervous system. From Vernetti et al. (92). Note the wide range of values in both the control and ASD groups. Eliciting anger, frustration or joy evoked a similar increase in sympathetic activity in the ASD and control group. In response to a fear-inducing stimulus, the control group exhibited an increase while the ASD group exhibited a decrease in sympathetic activity. However, the scatter plots show that most children with ASD and controls have a response that centers around 0, and most values in the ASD group fall within the range of control values. Hence a correct conclusion is that some children with ASD have a *decreased* arousal response to fear stimuli. The authors did not find a statistically significant difference in baseline sympathetic values and no correlation between the degree of sympathetic responses and the severity of autism. They conclude that toddlers with ASD should not be labeled as “dysregulated” or “upregulated” with respect to autonomic functioning.

Most studies using electrodermal activity do not support the theory of an overactive sympathetic system. Panju expected to find sympathetic hyperarousal and studied ASD children with high and low levels of anxiety; compared to a control group, ASD children with low levels of anxiety were not different from controls in baseline electrodermal activity nor in sympathetic responses to any stimulus (53). Contrary to their expectations, the baseline electrodermal activity in children with high anxiety levels was lower than controls. Levine et al. showed that in response to the Trier Social Stress Test, both ASD children and a control group showed a similar increase in sympathetic (electrodermal) activity (56). Toddlers with ASD displayed comparable electrodermal reactivity as typically developing peers in response to sensory stimuli in visual, auditory, tactile, and olfactory modalities as well as visual displays of repetitive movement (57). Joseph et al. showed normal baseline values. In measuring face recognition accuracy by skin conductance response, almost all children with ASD fell into the normal range with a few outliers (58). In an excellent study on assessing sympathetic activity in toddlers using skin conductance, where all the data were displayed in scatter plots (92), eliciting anger, frustration or joy evoked a similar increase in sympathetic activity in the ASD and control group (Figure 6). In response to a fear-inducing stimulus, the control group exhibited an *increase* while the ASD group exhibited a *decrease* in sympathetic activity, although the scatter plots show that most children with ASD and controls have a response that centers around 0 (Figure 6). Hence a correct conclusion appears that some children with ASD have a *decreased* sympathetic response to fear stimuli. The authors did not find a statistically significant difference in baseline sympathetic values and no correlation between the degree of sympathetic responses and the severity of autism. They conclude that toddlers with ASD should not be labeled as “dysregulated” or “upregulated” (92). However, unfortunately, they chose a title to their study that suggests the opposite. Bujnakova et al. (47) found lower values in children with ASD and suggested under-arousal, but discussed that this might reflect co-morbidities and not autism *per se*.

Muscatello et al. (30) studied the pre-ejection period, and no difference was found related to social tasks. However, the pre-ejection period is a measure of ventricular contractility and is not a good measure of general sympathetic functioning (59). For example, the pre-ejection period does not change with postural change from supine to standing, whereas it is well known that sympathetic activity markedly increases (59).

Using the Poincaré plot, no sympathetic differences in resting conditions, nor responses to a mental task, were found in adolescents with ASD compared to controls (35).

In summary, the overall conclusion must be that assessments of sympathetic activity in children with ASD using HRV measures or electrodermal activity do not support sympathetic autonomic dysfunction associated with autism. In response to tasks, the sympathetic activity goes up, similar to typically developing children, but there is no hyper-arousal.

## Effect of Medication on HRV

Almost all studies involving children with ASD are small, and given that there is a wide range of studied variables, this makes comparison and interpretation difficult. Mathewson et al. (60) measured RSA and heart rate to evaluate autonomic response to a challenging task, the Stroop test, dividing an adult ASD group based on the usage of antipsychotic medications. Controls were IQ-matched, and no subjects were intellectually impaired. The ASD-medication group had a significantly higher heart rate and lower RSA than both the control and ASD-no-medication group at baseline. Baseline RSA and heart rate were not significantly different between the control and ASD-no-medication groups. Contrary to their expectations, the autonomic responsiveness to the Stroop test was the same for the ASD and control group (60). Thapa et al. confirmed this in autistic children: no differences in heart rate, RMSSD, nor high-frequency power were observed in children not on psychotropic medication, compared to a control group (61). Hence, medication is likely a major factor in the higher heart rate found in ASD groups. A study involving 616 controls and 1,479 adults with anxiety showed that lower RSA in anxious subjects (52.1 vs. 45.1 ms; peak–valley method) survived adjustment for possible confounding factors as health indicators and lifestyle, but further adjustment for antidepressant use rendered all associations non-significant (62). Hence, drug use is a critical factor affecting HRV.

## Autism and Co-Morbidities

### Anxiety

Children with autism, on average, experience a higher level of anxiety than those without ASD (46, 63) however, anxiety is not a diagnostic criterium for autism spectrum disorder (2021). Anxiety can be a symptom, but it is difficult to differentiate the “appropriate” or “normal” level of anxiety to a stimulus experienced by a neurotypical person vs. someone with autism. It cannot be said that autism *causes* anxiety or vice versa; therefore, it is most appropriate to look at anxiety and autism independently.

A meta-analysis on the relationship between anxiety disorders and controls related to HRV parameters gave mixed results, with many studies finding on average a lower HRV in patients with anxiety disorders but many other studies finding no differences with controls (64).

All studies on ASD children are done, by definition, in experimental settings, and anxiety or stress will play a part in such studies in both controls and children with ASD. Studies on anxiety and autism give mixed conclusions, indicating that some but not all children with ASD have increased anxiety related to specific social stimuli (22, 65–68). Guy et al. reported that correlational analyses from their study indicated that low RSA was driven by factors that were part and perhaps entirely transdiagnostic— namely, symptoms of anxiety (22). Hence, some children with a high heart rate and/or low RSA may have, during the experimental conditions, a relatively high level of anxiety. An interesting study on autistic adults using self-reported frequency of autonomic nervous system-related physical health problems found that anxiety and stress but not autistic traits were correlated with autonomic dysfunction (69).

Studies in children with ASD always include stimuli that are thought to activate a stress response. But the response may just reflect the amount of physical activity and the accompanying metabolic demand (70). Koolhaas et al. suggest that a true stressor involves uncontrollability and unpredictability (70). It is suggested that a stress effect is more related to the recovery of a physiological response than the magnitude of the response (70). Children with ASD may be used to finding themselves in a situation that is not desirable, they may have found ways to adapt to it, and this may influence the response to a stimulus.

Parma et al. concluded that “ASD is related to reduced variability in basal sympathetic arousal and vagal modulation which can be taken as markers for inflexible responses” (71, 72). This conclusion is not consistent with their data. First, no responses were evaluated, only baseline values. There were no significant differences between control groups and patients with or without anxiety concerning baseline skin conductance, and there were no significant differences in the high-frequency component (RSA) between autistic children with and without anxiety and a control group with anxiety.

### Gastrointestinal Symptoms

Compared to typically developing children, there is a significantly higher prevalence of gastrointestinal symptoms in children with ASD (73), with functional constipation the most common gastrointestinal symptom. The autonomic nervous system plays a critical role in gut motility control. Vagal parasympathetic efferents provide parasympathetic innervation of the upper gastrointestinal tract, while sacral parasympathetic pathways innervate the distal gastrointestinal tract (74) to initiate propulsive contractile activity. Sympathetic nerves inhibit enteric cholinergic excitation to colonic smooth muscle and contract sphincters, contributing to decreased transit that may lead to constipation (75).

Ferguson et al. (76) investigated the relationship between autonomic nervous system activity and gastrointestinal symptoms in children with autism, finding a significant correlation between lower gastrointestinal tract symptoms, such as constipation, and lower parasympathetic tone. This supports the idea that, to a certain extent, lower gastrointestinal motility is controlled by parasympathetic activity (77). Parasympathetic activity at baseline was particularly strongly related to lower gastrointestinal symptoms in participants who reported a co-occurring anxiety disorder. Hence, a subgroup of ASD children may have a low RSA due to gastrointestinal symptoms.

## Does Measuring HRV Parameters in Children with ASD Have Relevance?

Based on heart rate and RSA data, most children with autism do not display autonomic dysfunction. Hence, these parameters should not be studied to learn more about the pathophysiology underlying the symptoms related to the diagnostic criteria of autism. However, if a better understanding of co-morbidities with autonomic dysfunction is to be obtained, it may be worthwhile to study HRV parameters.

### How Should HRV Be Measured?

When autonomic functioning is to be assessed in patients with ASD, it is not advisable to only pay attention to RSA, just because it is the focus of the polyvagal theory. A comprehensive assessment should include other parameters as outlined by task forces (78). This should include the Baevsky stress index (13, 59, 79) for sympathetic function. Recently, the Baevsky index has been shown to be a reliable measure of sympathetic activity in the active standing test (77). Beversdorf reviewed the role of adrenergic antagonists in ASD treatment, and evaluation of their potential use may rely on HRV assessment as well as plasma catecholamine levels (80). Some parameters of HRV can be graphically captured using the Poincaré plot. The Poincaré plot is primarily a non-linear technique, but the most often used descriptors SD1 and SD2 are measuring linear aspects of the heartbeat intervals; they do not add value to existing HRV indexes (81). In particular SD1, which is mathematically equivalent to RMSSD; RMSSD = √2 × SD1 (82). SD2 is also highly correlated with RMSSD and should therefore not be used as a sympathetic descriptor (82, 83). It is important to note that if HRV parameters are used to study co-morbidities with autonomic dysfunction, one has to account for covariates that affect autonomic function such as age, sex, body posture at the time of recording, and time of day of the recording (25). Several studies provide evidence that HRV parameters are different for children who are intellectually impaired (IQ < 70) compared to non-intellectually impaired children (IQ ≥ 70) (8). However, the appropriateness of subdividing children according to IQ is questionable since intelligence can be expressed and measured in various ways.

### Long-Term Measurements

In addition to the short-term HRV parameters to measure baseline and response to stimuli, long-term 24 h HRV analysis may reveal important information. Not so much average HRV parameters over 24 h since this is very much dependent on activities performed during the day and night, but analysis of events and analysis of HRV during sleep (84). Children with ASD frequently suffer from difficulties falling asleep or nightmares (85, 86). Research is ongoing to examine HRV descriptors for dynamic changes over time that are not captured by the classic short-term parameters (84). Long-term assessments have the additional advantage of being implemented in a familiar setting.

### Reporting Absolute Values

of reporting absolute values of HRV parameters in the ASD literature is common but makes it difficult to compare studies and relate the findings to overall control values. In some studies, neither absolute values are reported nor comparisons to control values (87). Any RSA response should also be related to the absolute value at baseline. It is possible that when the hypothesis is tested whether a stimulus is increasing the RSA, a subject with an RSA of 9 ln(ms^2^) is less likely to respond in this way compared to a subject with an RSA of 5 ln(ms^2^), because in the first, the parasympathetic nervous system may be in a heightened state of arousal. It is also important to report all individual values. This will show “outliers” that might be clinically highly relevant. In this respect, the bar graphs we present here may not reflect an exact distribution in that they suggest an even distribution of values, but the individual values are not shown in the reported studies. What is needed is knowledge of the distribution of values, hence the reporting of all absolute values in scatter plots. Concerning RSA obtained from HF power, it may be worthwhile to show the distribution of HF power, since RSA is a logarithmic transformation of HF power and as such (designed to) diminish outliers. If data are too extensive to be put in the publication, they can be expressed as violin plots or submitted as Supplementary Material.

### What Are Suitable Control Values?

HRV parameters have a wide range of control values and are very susceptible to circumstances, such as simple body movements. Harteveld et al. studied control values in 4,822 children and concluded that the wide range of normal values would cause problems of interpretation for clinical studies (25). A very small group of typically developing children does not give us a true normal range of control baseline values, assumed to represent general parasympathetic health. A study investigating changes in response to stimuli obviously needs a control group. Comparing the changes in response to stimuli is likely more relevant than focusing on the absolute values in such studies. Responses should also be related to the baseline absolute values.

### Mentioning Units of Measurement

It is common in the ASD-HRV literature not to mention units for HRV parameters. This is unfortunate since this makes comparisons and interpretations by others difficult. For example, numerical values are given to the term “HRV”, but the definition of HRV as a specific autonomic function parameter is variable. Sometimes it is used as equivalent to RMSSD and sometimes used as equivalent to RSA. Sometimes absolute values are used and sometimes logarithmic transformations. RSA is most often derived from the power of the high-frequency range of inter-beat intervals, but sometimes it is measured by subtracting the shortest inter-beat interval during inhalation from the longest inter-beat interval during exhalation. The high-frequency range is most often, but not always, defined as 0.15–0.40 Hz, equivalent to the normal breathing frequency range of 8–25 breaths per min. Sometimes the term “normalized units” is used, but it is often not explained how the data are normalized. This results in difficulties of interpretation and may result in inaccurate comparisons between studies. For RSA, it is best first to report the raw values of HF power and determine the distribution of values since we are emphasizing the recognition of subgroups within an ASD cohort, and it should be noted that the logarithmic transformation from HF power to RSA may obscure “outliers”; it is in fact designed to “normalize” the data, but the “outliers” may constitute a clinically significant subgroup.

HRV studies are always time-consuming and involve patient and parent time. It is, therefore, unfortunate that usually only RSA is measured, no doubt because of its emphasis within the polyvagal theory. To get an optimal picture of HRV, all possible parameters should be reported, including the Baevski index derived from heartbeat intervals.

## The Polyvagal Theory and ASD

The polyvagal theory proposes that children with ASD have chronic sympathetic activation or a chronically mobilized state (8), but studies with electrodermal activity do not support this. Electrodermal activity does register sympathetic responses to social activities, but abnormal excitation is not seen. Another proposed measure of chronic sympathetic activation is elevated heart rate. Porges et al. make strong statements that fail scrutiny, such as: “In particular, children and adolescents aged 8–18 years with ASD and intellectual impairment have a heart rate that is 20 beats per min higher than typically developing controls” (8) with reference to Goodwin et al. (2006). This sounds like a definitive statement about ASD; however, Goodwin et al. report on five children with ASD and five children with normal development, with one normal child having a heart rate of 50 bpm; hence this can hardly be accepted as a definitive statement on ASD. Excess excitation of the sympathetic nervous system is central to the polyvagal autonomic dysfunction theory. The tasks that ASD children are asked to do are designed to emphasize the typical events that the children are deemed to have difficulty with due to ASD. These tasks increase sympathetic activity, as shown by electrodermal activity. Hence the conditions appear to be perfect for showing exaggerated sympathetic arousal, but it is not observed.

The polyvagal theory proposes that children with autism show vagal withdrawal or “chronic mobilization” that would lead to low baseline RSA. RSA is integral to the polyvagal theory. It is proposed to reflect autonomic activity from the nucleus ambiguous involved in heart rate regulation, breathing, and responses involving emotion. Most children with ASD have a baseline RSA that is the same as controls, and there is no evidence that the absolute baseline value of RSA in children with ASD can be considered a sign of autonomic dysfunction. Furthermore, Porges et al. (5) propose the idea that children with ASD are unable to display “appropriate” psychophysiological flexibility in response to stimuli due to autonomic inflexibility. Expressly, children with ASD may be at one particular autonomic “setting” and are not able to demonstrate psychophysiological flexibility to stimuli (e.g., appropriate vagal withdrawal to attention-demanding stimuli) (8). Most studies do not support abnormal RSA responsiveness in children with ASD, which means that either this neural communication involving the nucleus ambiguous is normal in most children with ASD or that RSA is not the best window to observe relevant autonomic functioning in ASD. It is acknowledged that RSA is but one of the windows into autonomic functioning and that other brain areas such as the amygdala and its effect on the functioning of the nucleus ambiguous deserve our attention to understand the physiology behind the behaviors of children with ASD (8, 35, 88). Furthermore, the hypothalamic pituitary adrenocortical axis and the adrenal medulla are critical players in the response to (potential) stress stimuli in metabolic and cardiovascular preparation of the body to perform behavior (70, 89).

## Perspective

In many studies with ASD children, statistical differences in HRV parameters are uncritically deemed to be clinically relevant. Children with ASD are categorized as having inhibited parasympathetic activation and being in a state of chronic vagal withdrawal and heightened sympathetic arousal based on statistical differences between groups that may have no clinical importance. Based on generally accepted control values, almost all children with ASD have a normal heart rate and a normal RSA at baseline, and most children also fall within the control values of the study control group. Hence, there is no evidence that baseline HRV values in children with ASD point to autonomic dysfunction underlying the typical symptoms that define ASD. The autonomic nervous system plays a vital role in children's adjustment to physical stimuli such as standing up, walking, change in outside temperature etc. It is assumed that this is also true for social or emotional stimuli and would be measurable by HRV assessment. Children with ASD often have an atypical reaction to provocations; hence, evaluating autonomic functioning in response to such stimuli would be logical. A stressful social interaction, similar to standing up, usually increases heart rate and decreases RSA (e.g., Figure 2). The fact is that in most studies, the autonomic nervous system of children with ASD reacts to social stimuli in this normal manner, very similar to control groups. Interestingly, when RSA goes up in response to a positive, relaxing intervention, it does so similarly in controls and children with ASD (30). A recent review by Benevides and Lane (19) also concluded that no apparent differences in resting parasympathetic activity emerged from the literature, nor differences in task-related ANS activity (19). The *data* from almost all HRV studies on ASD herald the positive news that there is no evidence of general autonomic dysfunction associated with the typical ASD traits. It is disheartening that most *conclusions* stated in these studies suggest the opposite.

This leaves us with the finding that some children with ASD have a baseline value or a reaction to stimuli that fall outside of the “normal range” being defined as within the 95% confidence interval or a value of <1 SD away from the mean. It is supported by many studies that a relatively low RSA or a relatively high heart rate is associated with a comorbidity that is related to autonomic dysfunction, such as anxiety or gastrointestinal motor dysfunctions. Hence, it may be worthwhile to do HRV analysis to identify those children with ASD suspected of having underlying autonomic conditions, e.g., a low RSA may benefit from exercise training to promote healthy parasympathetic reactivity (90, 91).

## Data Availability Statement

The original contributions presented in the study are included in the article/Supplementary Material, further inquiries can be directed to the corresponding author.

## Author Contributions

All authors listed have made a substantial, direct, and intellectual contribution to the work and approved it for publication.

## Funding

Financial support was received from the Canadian Institutes of Health Research.

## Conflict of Interest

The authors declare that the research was conducted in the absence of any commercial or financial relationships that could be construed as a potential conflict of interest.

## Publisher's Note

All claims expressed in this article are solely those of the authors and do not necessarily represent those of their affiliated organizations, or those of the publisher, the editors and the reviewers. Any product that may be evaluated in this article, or claim that may be made by its manufacturer, is not guaranteed or endorsed by the publisher.

## Abbreviations

ASD: Autism spectrum disorder
ANS: Autonomic nervous system
HRV: Heart rate variability
RSA: Respiratory sinus arrhythmia
RMSSD: root mean square of successive differences between heartbeats
bpm: beats per minute.
