# Supplementary figures and images for: Autism Spectrum Disorder in Children Is Not Associated With Abnormal Autonomic Nervous System Function: Hypothesis and Theory
Source: Front Psychiatry. 2022 Mar 15;13:830234. doi: 10.3389/fpsyt.2022.830234 (PMC8964964; doi:10.3389/fpsyt.2022.830234)

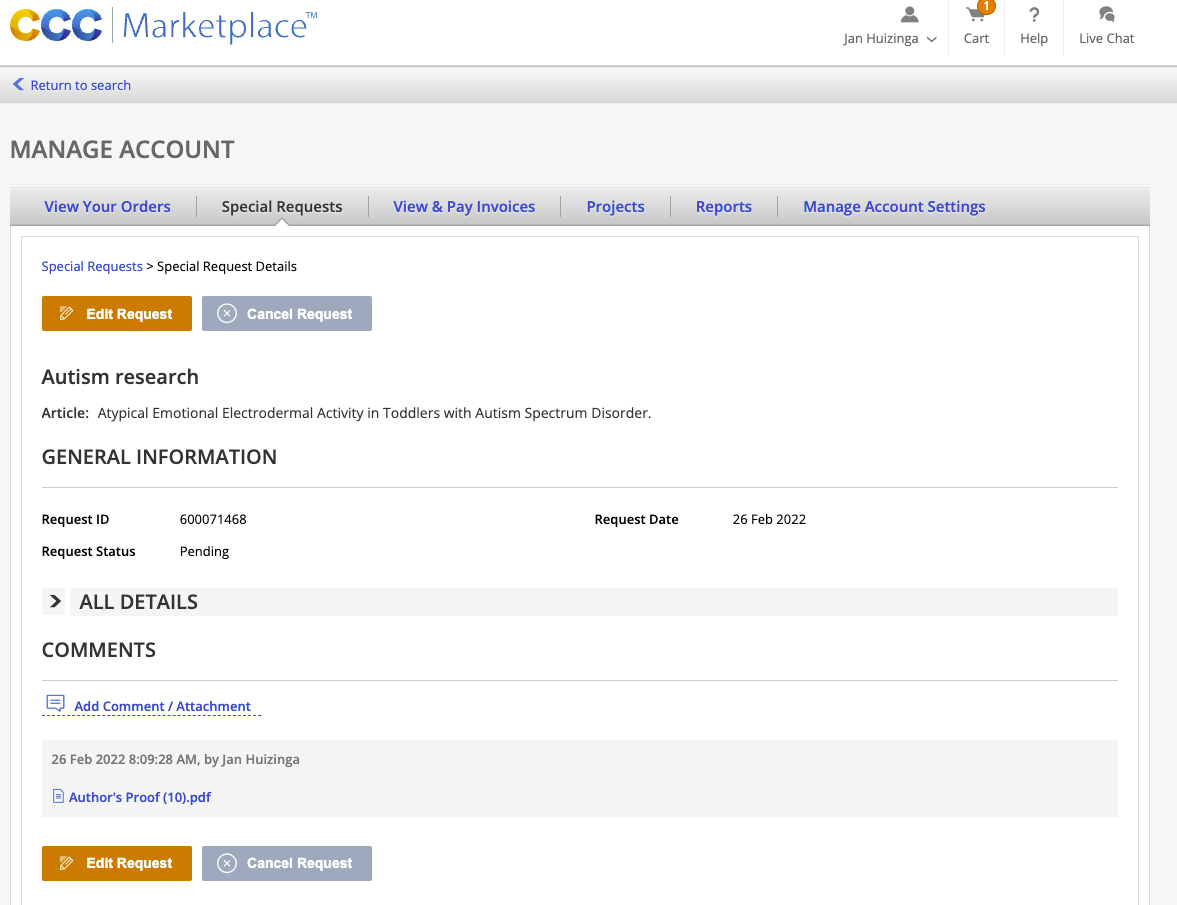

Supplement: Supplementary file 1 [file Data_Sheet_1.docx]
