# Supplementary material for: Autism Spectrum Disorder in Children Is Not Associated With Abnormal Autonomic Nervous System Function: Hypothesis and Theory
Source: Front Psychiatry. 2022 Mar 15;13:830234. doi: 10.3389/fpsyt.2022.830234 (PMC8964964; doi:10.3389/fpsyt.2022.830234)
Supplement: Supplementary file 2 [file Data_Sheet_2.docx]

✷ Author names - Complete, accurate and consistent with your previous publications.

Yes

✷ Afliations - Complete and accurate. Follow this style when applicable: Department, Institute, University, City, Country.

Yes

✷ Tables - Make sure our formatting style did not change the meaning/alignment of your Tables.

OK

✷ Figures - Make sure we are using the latest versions.

OK

✷ Funding and Acknowledgments - List all relevant funders and acknowledgments.

OK

✷ Conict of Interest - Ensure any relevant conflicts are declared.

Yes

✷ Supplementary les - Ensure the latest files are published and that no line numbers and tracked changes are visible. Also, the supplementary files should be cited in the article body text.

No supplementary files

✷ Queries - Reply to all typesetters queries below.

Yes

✷ Content - Read all content carefully and ensure any necessary corrections are made. Author Queries Form Query No. Details Required Author’s Response

**Q1 The citation and surnames of all of the authors have been highlighted. Check that they are correct and consistent with the authors’ previous publications, and correct if need be. Please note that this may affect the indexing of your article in repositories such as PubMed.**

Correct

**Q2 Confirm whether the insertion of the article title is correct.**

No, there should NOT be a comma after Hypothesis

**Q3 Confirm that all author affiliations are correctly listed. Note that affiliations are listed sequentially as per journal style and requests for non-sequential listing will not be applied. Note that affiliations should reflect those at the time during which the work was undertaken.**

Correct

**Q4 Confirm that the email address in your correspondence section is accurate. Please note that any changes to the corresponding authorship would require individual confirmation from all original and added/removed corresponding authors.**

Correct

**Q5 Confirm that the keywords are correct and keep them to a maximum of eight and a minimum of five. (Note: a keyword can be comprised of one or more words.) Note that we have used the keywords provided at Submission. If this is not the latest version, please let us know.**

Correct

**Q6 If you decide to use previously published, copyrighted figures in your article, please keep in mind that it is your responsibility, as the author, to obtain the appropriate permissions and licenses and to follow any citation instructions requested by third-party rights holders. If obtaining the reproduction rights involves the payment of a fee, these charges are to be paid by the authors.**

**I have included one permission and another permission is pending, see attached files**

**Q7 Check if the section headers (i.e., section leveling) were correctly captured.**

Yes

**Q8 Confirm that the short running title (top right corner starting from the 2 nd page) is correct, making sure to keep it to a maximum of five words.**

Please shorten and change to: Autonomic Functioning in Autism

**Q9 Ensure that all the figures, tables and captions are correct, and that all figures are of the highest quality/resolution. Please note that Figures and Tables must be cited sequentially, as per section 2.2 of the author guidelines.**

Correct

**Q10 Verify that all the equations and special characters are displayed correctly.**

Correct

**Q11 The caption of Table 1 is missing and must be provided.**

Please add caption: RSA control values in healthy children

**Q12 Confirm that the Data Availability statement is accurate. Note that we have used the statement provided at Submission. If this is not the latest version, please let us know.**

OK

**Q13 The author contribution section is mandatory and a standard statement has been inserted. Please edit as needed to accurately reflect the contribution of each author.**

Correct

**Q14 We have replaced the section head “Acknowledgments” with “Funding.” Please confirm that this is correct.**

No, this is not correct. We have accepted the section head FUNDING and added the grant number. But this has to be followed by ACKNOWLEDGEMENT and under this subheading it should be mentioned: AB is an Autism Support Associate

**Q15 Ensure to add all grant numbers and funding information, as after publication this will no longer be possible. All funders should be credited and all grant numbers should be correctly included in this section.**

Done

**Q16 Your article has no Supplementary Material; however, there are citations for “Supplementary Material”. Could you clarify this?**

We have deleted the words Supplementary Material

**Q17 Provide the complete details for the following references. “Lau et al., 2020; Goodwin et al., 2006; Porges, 1976.”**

Ashley: this is a bit of a problem. We cannot include new references, since that would screw up all other references since they are numbered. So we need to change the reference into an existing one with a number that is mentioned earlier or delete it.

We have removed Lau et al. The reference is not necessary

We have removed Porges 1976 since the correct reference is at the end of this sentence.

Goodwin et al. 2006. This reference is essential and we need to include it. We indicate the reference in the proof.

**Q18 Provide the city name for “(2).”**

Changed to webpage and accession date

**Q19 Please provide last accession date for “https://www.cdc.gov/ncbddd/autism/hcp--dsm.html”.**

Done

**Q20 Provide the volume number for “(13).”**

Done

**Q21 Provide the volume number, page range, and doi for “(59).”**

Info provided, no volume number exists

**Q22 Provide the doi for the following references. “(39, 45, 79).”**

Indicated in proof. PLEASE REPLACE 79 with the reference indicated in the proof

**Q23 Confirm if the text included in the Conflict of Interest statement is correct.**

Correct

**Q24 Figures 5, 6 has not been mentioned in the article. Please add a citation within the text, noting that Figures must appear in sequence.**

Citation to Figures 5,6 have been added to lines 851 & 1088, respectively.

**Q25 Check that the amendments to the title are fine.**

There should not be a comma following “Hypothesis” in the title.
