# Supplementary material for: Autism Spectrum Disorder in Children Is Not Associated With Abnormal Autonomic Nervous System Function: Hypothesis and Theory
Source: Front Psychiatry. 2022 Mar 15;13:830234. doi: 10.3389/fpsyt.2022.830234 (PMC8964964; doi:10.3389/fpsyt.2022.830234)
Supplement: Supplementary file 3 [file Image_1.pdf]

SPRINGER NATURE

Functional autonomic nervous system profile in children with autism spectrum disorder

Author: Azadeh Kushki et al
Publication: Molecular Autism
Publisher: Springer Nature
Date: Jul 4, 2014

Copyright © 2014, Kushki et al.; licensee BioMed Central Ltd.

Creative Commons

The request you have made is considered to be non-commercial/educational. As the article you have requested has been distributed under a Creative Commons license (Attribution-Noncommercial), you may reuse this material for non-commercial/educational purposes without obtaining additional permission from Springer Nature, providing that the author and the original source of publication are fully acknowledged (please see the article itself for the license version number). You may reuse this material without obtaining permission from Springer Nature, providing that the author and the original source of publication are fully acknowledged, as per the terms of the license. For license terms, please see http://creativecommons.org/

BACK | CLOSE WINDOW

© 2021 Copyright - All Rights Reserved | Copyright Clearance Center, Inc. | Privacy statement | Terms and Conditions
Comments? We would like to hear from you. E-mail us at customercare@copyright.com
